# The Prodomain-bound Form of Bone Morphogenetic Protein 10 Is Biologically Active on Endothelial Cells*

**DOI:** 10.1074/jbc.M115.683292

**Published:** 2015-12-02

**Authors:** He Jiang, Richard M. Salmon, Paul D. Upton, Zhenquan Wei, Aleksandra Lawera, Anthony P. Davenport, Nicholas W. Morrell, Wei Li

**Affiliations:** From the Department of Medicine, University of Cambridge School of Clinical Medicine, Addenbrooke's Hospital, Hills Road, Cambridge, CB2 0QQ United Kingdom

**Keywords:** bone morphogenetic protein (BMP), cell biology, endothelial cell, signal transduction, transforming growth factor beta (TGF-β), bone morphogenetic protein 10 (BMP10)

## Abstract

BMP10 is highly expressed in the developing heart and plays essential roles in cardiogenesis. BMP10 deletion in mice results in embryonic lethality because of impaired cardiac development. In adults, BMP10 expression is restricted to the right atrium, though ventricular hypertrophy is accompanied by increased BMP10 expression in a rat hypertension model. However, reports of BMP10 activity in the circulation are inconclusive. In particular, it is not known whether *in vivo* secreted BMP10 is active or whether additional factors are required to achieve its bioactivity. It has been shown that high-affinity binding of the BMP10 prodomain to the mature ligand inhibits BMP10 signaling activity in C2C12 cells, and it was proposed that prodomain-bound BMP10 (pBMP10) complex is latent. In this study, we demonstrated that the BMP10 prodomain did not inhibit BMP10 signaling activity in multiple endothelial cells, and that recombinant human pBMP10 complex, expressed in mammalian cells and purified under native conditions, was fully active. In addition, both BMP10 in human plasma and BMP10 secreted from the mouse right atrium were fully active. Finally, we confirmed that active BMP10 secreted from mouse right atrium was in the prodomain-bound form. Our data suggest that circulating BMP10 in adults is fully active and that the reported vascular quiescence function of BMP10 *in vivo* is due to the direct activity of pBMP10 and does not require an additional activation step. Moreover, being an active ligand, recombinant pBMP10 may have therapeutic potential as an endothelial-selective BMP ligand, in conditions characterized by loss of BMP9/10 signaling.

## Introduction

Bone morphogenetic protein 10 (BMP10)^7^ plays an important role in embryonic cardiac development. During cardiogenesis, BMP10 is exclusively expressed in the ventricular trabecular myocardium (1) of mouse embryos at E9.0–13.5, a critical time for cardiac growth and chamber maturation. The hearts from *BMP10*^−/−^ mice, which die during embryogenesis, show hypoplastic and thin ventricles with little trabeculation (2). Overexpression of BMP10 in the postnatal myocardium leads to smaller hearts within 6 weeks after birth, significant reduction in ventricular chamber dimensions, thickening of the ventricular wall, and narrowing in the subaortic region (3), indicating a role for BMP10 in postnatal cardiomyocyte hypertrophic growth. In adults, BMP10 expression is restricted to the right atrium (2) and its role in cardiac physiology is not known. However, increased BMP10 expression was observed in the hypertrophied ventricles in a rat model of hypertension (4). Such restricted and highly regulated expression of BMP10 in adult hearts strongly suggests that it plays an important role in physiology. To better define the function of BMP10 *in vivo*, it is essential to identify whether the atrium-secreted BMP10 is fully active or under additional regulation at the protein level.

BMP10 is a member of the transforming growth factor β (TGFβ) superfamily. These ligands initiate cellular signaling by forming a signaling complex comprising two type I and two type II receptors, both of which are serine/threonine kinases. Upon signaling complex formation, the type I receptor is activated due to the phosphorylation by the constitutively active type II receptor, and phosphorylates the downstream transcription factors to control gene expression. There are five type I receptors: activin receptor-like kinase 1 (ALK1), ALK2, ALK3, ALK6, and ALK7, and three type II receptors: BMP receptor type II (BMPR-II), activin receptor type IIA (ActR-IIA), and ActR-IIB, that mediate BMP signaling. BMP9 and BMP10 belong to the same subgroup of the TGFβ superfamily based on amino acid sequence similarities (5), and they are also the only two known ligands that can specifically activate ALK1. The ALK1-mediated signaling pathway plays important roles in normal and tumor angiogenesis and ALK1-Fc (Dalantercept, ALK1 extracellular domain-Fc fusion protein acting as a ligand trap) is currently in clinical trials for treating solid tumors (6). Since ALK1 is almost exclusively expressed on vascular endothelial cells (7), BMP9 and BMP10 signaling pathways are likely to play important roles in endothelial cell biology.

TGFβ/BMP family ligands are synthesized as the pre-proprotein and processed into the prodomain and mature growth factor domain (GFD). Two major roles have been described for the prodomains. First, they interact with fibrillin-1 or other extracellular matrix components to target the growth factors to the specific extracellular space (8). Secondly, the prodomain can regulate the activity of the mature growth factor by either conferring latency to the growth factor (9, 10), or not interfering with growth factor activity (8). For example, prodomain-bound TGFβ and myostatin exist in latent forms and require further mechanisms to activate their signaling activity, such as the proteolytic cleavage of prodomain by BMP1 (10) or integrin-dependent activation (11). In contrast, the prodomains of BMP4, 5, and 7 have been shown to bind to their cognate GFD with very high affinities, but do not affect their signaling activity (8). Interestingly, using a mouse C2C12 myoblast cell-based activity assay with inhibitor of DNA-binding 3 (ID3) gene induction as a readout, the prodomain from BMP10 has been shown to potently inhibit BMP10 activity, and it was thus proposed that the pBMP10 complex might be similar to TGFβ and myostatin in that it is latent and requires additional factors to achieve activation (8). Apparently consistent with this observation, BMP10 GFD protein has been detected in human and mouse sera by ELISA (12) and proteomic approaches (13). Of note, two independent groups were unable to detect circulating BMP10 activity in human serum (14, 15), although BMP10 activity was recently identified in mouse serum (16). This controversy on circulating BMP10 activity needs to be resolved since it is difficult to study BMP10 function *in vivo* without knowing whether there are additional activation mechanisms involved.

Extensive studies on BMP9 have been reported in the past decade. It has been shown that BMP9 is a vascular quiescence factor, circulating at active concentrations, which inhibits endothelial cell proliferation and VEGF-induced angiogenesis (14, 17, 18). Pathogenic mutations in ALK1 which cause hereditary hemorrhagic telangiectasia type 2 result in defective BMP9 signaling (19). In contrast, studies on BMP10 are more limited, partially because its activity has not been consistently detected in human serum or plasma. Interestingly, using BMP10 GFD, *in vitro* cell biology studies show that BMP10 regulates a similar set of genes to BMP9, and with similar potency (12, 20). More intriguingly, *BMP9* null mice are viable and it has been proposed that BMP9 and BMP10 can mediate functionally redundant signals *in vivo* and BMP10 can substitute BMP9 in postnatal retinal vascular remodeling (12). In contrast, BMP9 cannot replace BMP10 in cardiac development even when it is expressed under a BMP10 promoter, indicating a unique signaling capacity of BMP10 in cardiac development (16).

To compensate for BMP9 function in *BMP9*^−/−^ mice, circulating BMP10 would need to be active. This implies that either circulating BMP10 is active, or an additional BMP10 activation mechanism is in place in the *BMP9*^−/−^ mice. Whether the pBMP10 complex is latent or biologically active is directly relevant to this question. More importantly, if pBMP10 is not latent, the high level expression of BMP10 in the adult right atrium might indicate an important physiological role for BMP10 in the adult heart that is yet to be identified. In addition, whether pBMP10 is active or not is of significant translational interest. For example, based on the observation that the prodomain is inhibitory, it has been proposed that the BMP10 prodomain could be developed into a ligand trap for anti-tumor therapy (8). On the other hand, if pBMP10 itself is active, it might have therapeutic potential, because administration of the ALK1-specific ligand, BMP9, has been shown to be beneficial in preclinical models of pulmonary arterial hypertension (PAH) (21).

In this report, we showed that the prodomain of BMP10 did not inhibit its activity in multiple endothelial cell lines. In addition, recombinant human pBMP10 expressed in mammalian cells and purified under native conditions was fully active, despite pBMP10 being a very stable complex. Furthermore, we provided evidence that BMP10 derived from endogenous sources, including cultured mouse right atrium or human plasma, was fully active on endothelial cells. Finally, we demonstrated that active BMP10 secreted from the mouse right atrium was the prodomain-bound complex.

## Experimental Procedures

### 

#### 

##### Materials

Anti-BMP9 antibody (MAB3209), anti-BMP10 antibody (MAB2926), BMP10 prodomain (3956-BP-050), anti-BMP10 propeptide antibody (AF3956), biotinylated anti-BMP10 propeptide antibody (BAF3956), ALK1-Fc (370-AL), human BMP10 GFD (2926-BP-025) were all purchased from R&D Systems, Inc. Anti-phosphoSmad1/5/8 and anti-phosphoSmad1/5 antibody were purchased from Cell Signaling Technology. Anti-ID1 (M085) and anti-ID3 (M100) antibodies were purchased from CalBioreagents (San Mateo, CA). HiTrap Q FF and Superdex 200 10/30 columns were purchased from GE Healthcare. Human pulmonary artery endothelial cells (HPAECs) and endothelial growth medium were purchased from Lonza, UK. Human aortic endothelial cells (HAECs) were purchased from PromoCell. All other tissue culture medium were purchased from Life Technologies. All plasmid and RNA purification kits were purchased from Qiagen. The Gel Filtration Calibration Kit was purchased from Sigma-Aldrich.

##### BMP10 Prodomain Inhibition Assay

Because of different cells having different sensitivities to BMP10, 10 ng/ml of BMP10 was used to treat C2C12 mouse myoblasts and 1 ng/ml used for endothelial cells. BMP10 GFD was pre-incubated with a 4-fold increased molar ratio of BMP10 prodomain with 0.5% (*v*/*v*) BSA as a carrier protein. The mixture was incubated for 2 h at room temperature before being added dropwise onto serum-starved cells. Cells were snap-frozen after 1 h of treatment and harvested in SDS-lysis buffer for subsequent immunoblot analysis as described previously (22).

##### Expression and Purification of pBMP10

Human full-length *proBMP10* cDNA was cloned into pCEP4 between XhoI and BamHI sites and verified by DNA sequencing. Plasmids containing *proBMP10* were transfected into HEK EBNA cells using polyethylenimine as described previously (22). To facilitate processing, human full-length furin cDNA, cloned in the same vector, was co-transfected. To purify pBMP10, 5 liters of conditioned medium were loaded onto a 100 ml of Q Sepharose column, pre-equilibrated in 20 mm Tris·HCl, pH 7.4, and bound proteins were washed and eluted using NaCl gradients from 100 mm to 2 m. After another step of Q-Sepharose high performance column separation, fractions containing pBMP10 were pooled, concentrated in a VivaSpin column, and loaded onto a HiLoad 16/600 Superdex 200 pg column pre-equilibrated in 20 mm Tris·HCl, pH 7.4, 150 mm NaCl. Fractions containing pBMP10 were dialyzed into 20 mm Tris·HCl, pH 7.8, 25 mm NaCl and further purified on a MonoP 5/200 GL column pre-equilibrated in 20 mm Tris·HCl, pH 7.8. A final Superdex 200 column, pre-equilibrated in 150 mm NaCl, 20 mm Tris·HCl, pH 7.4, was used to separate the pBMP10 from excess prodomain.

##### Quantification of pBMP10

To compare the activity of in-house purified pBMP10 with the commercial BMP10 GFD from R&D Systems, pBMP10 was quantified as the concentration of mature BMP10 GFD in two steps. In the initial step, pBMP10 was quantified by Coomassie Blue staining on an SDS-PAGE using BSA as a standard. The result of this initial quantification was used as a guide to prepare the samples in the second round of quantification using immunoblotting and commercial BMP10 GFD as a standard. The concentrations of pBMP10 in all the cell assays described here refer to the concentrations of mature GFD in the pBMP10 complex.

##### Expression and Purification of BMPR-II Extracellular Domain (ECD)

Human BMPR2 (NM_001204) ECD, containing residues 27–150, was cloned into pET39b (Novagen) between NcoI and NotI sites to generate a construct expressing DsbA-(His)_6_-BMPR2 ECD fusion protein. A TEV protease (Tobacco Etch Virus nuclear inclusion A endopeptidase) cleavage site was introduced at the N terminus of BMPR2 ECD to facilitate the cleavage of the fusion protein. The insert was confirmed by DNA sequencing. The plasmid was transformed into bacterial strain Rosetta DE3 for protein expression. In brief, cells were grown at 37 °C until mid-log phase followed by isopropyl-β-d-thiogalactoside induction at 25 °C overnight. Total proteins in the periplasmic compartment were extracted following the pET System Manual (Novagen) and applied to a 5 ml of nickel-nitrilotriacetic acid column (GE Healthcare). Fractions containing the fusion protein were pooled, dialyzed into TBS, and incubated with His-tagged TEV protease overnight before being loaded again onto a precharged nickel-nitrilotriacetic acid column to remove the DsbA tag and TEV protease. BMPR-II ECD, which was in the flowthrough of the column, was concentrated and further purified on a S75 gel filtration chromatography. The final BMPR-II is over 99% pure on an SDS-PAGE (data not shown).

##### Human Heart Tissues

Surgical samples of tissues were obtained from normal hearts not suitable for further transplantation with informed consent from the Papworth Hospital Research Tissue Bank and experiments performed with local ethical approval (REC 05/Q0104/142).

##### Ex Vivo Culture of Mouse Atria

Mouse left atrium (LA) and right atrium (RA) were dissected from adult C57BL/6 mice (12–16 weeks old) and either snap-frozen in liquid nitrogen for RNA extraction or kept in ice-cold PBS for *ex vivo* cultures. All animal procedures in this study were carried out according to Home Office regulations and approved by the local animal care committee. After two additional washing steps with PBS, pooled fresh atria were transferred into T25 flasks containing DMEM (0.8 ml medium per atrium, LA and RA from the same mouse were cultured in parallel) supplemented with Antibiotic-Antimycotic (Life Technologies). The *ex vivo* culture were kept at 37 °C with 5% CO_2_. Conditioned medium was harvested between 48 to 72 h and BMP activity in the conditioned medium was evaluated in the endothelial cell signaling assays in the presence or absence of anti-BMP9 antibody, anti-BMP10 antibody, or ALK1-Fc, respectively.

##### RNA Extraction and Quantitative PCR (Q-PCR)

Cells were grown in 6-well plates and serum-restricted overnight prior to treatments. At the end of time courses, cells were washed with PBS and snap-frozen on dry ice. Extraction of total RNA was performed using RNeasy Plus Mini Kit (Qiagen). Total RNA from mouse atrium was extracted using RNeasy Fibrous Tissue Mini Kit (Qiagen). Reverse transcription was carried out following the manufacturer's instructions (High-Capacity cDNA Reverse Transcription Kit, Applied Biosystems). Q-PCR reactions were prepared with 4.5 μl of diluted cDNA samples and 10.5 μl of master mix containing 7.5 μl of the SYBR Green Jumpstart Taq ReadyMix (Sigma-Aldrich), 200 nm of each forward and reverse primers and 10 nm of Rox Reference Dye. The following forward and reverse primers were used to amplify the corresponding human genes: *ID1*: 5′-CTGCTCTACGACATGAACGGC, 5′-TGACGTGCTGGAGAATCTCCA; β*-2-microglobulin (B2M):* 5′-CTCGCGCTACTCTCTCTTTCT, 5′-CATTCTCTGCTGGATGACGTG; *BMPR2*: 5′-CAAATCTGTGAGCCCAACAGTCAA, 5′-GAGGAAGAATAATCTGGATAAGGACCAAT. QuantiTect Q-PCR primers for human BMP10 (Hs_BMP10_1_SG), mouse *BMP9* (Mm_Gdf2_1_SG), *BMP10* (Mm_Bmp10_1_SG) and *B2M* (Mm_B2m_2_SG) were purchased from Qiagen. Reactions were amplified on a StepOnePlus^TM^thermal cycler (Applied Biosystems) and the relative expression of target mRNAs was normalized to *B2M* using the ΔΔCT method, expressed as the fold-change relative to control samples, and shown as mean ± S.E. where applicable.

##### Fractionation of Conditioned Medium from RA

A Superdex 200 10/300 GL column was equilibrated in PBS and proteins of known molecular weights from the Gel Filtration Calibration Kit were fractionated to obtain the elution volumes for proteins of different sizes. Conditioned medium from RA or diluted in-house purified pBMP10 were passed though the same column under identical conditions, and the eluate was collected as 250-μl fractions after the void volume. Every second fraction was assessed for BMP10-activity using BMP-responsive element (BRE)-Luciferase Assay in C2C12 cells as described previously (22) with the following modifications: C2C12 cells were seeded in 24-well plate at 3 × 10^4^ cells/well. All wells were transfected with 400 ng of plasmid containing BRE-luciferase (kind gift from Professor P. ten Dijke), 40 ng of *Renilla* luciferase, and 5 ng of pcDNA3:hALK1 per well. Cells were quiesced for 16 h in serum-free DMEM and then treated in triplicate for 6 h with an appropriate dilution of each fraction. Firefly and *Renilla* luciferase activities were measured and analyzed as described previously.

##### pBMP10 ELISA

A high binding 96-well ELISA plate was coated with anti-BMP10 monoclonal antibody (2 μg/ml) in PBS at 4 °C overnight. After washing with PBS, wells were blocked with 1% BSA in PBS for 2 h, washed with PBS, and 100 μl of undiluted fractions were applied in duplicate. After 2 h of incubation at room temperature, wells were washed again and probed with biotinylated anti-BMP10 propeptide antibody (0.4 μg/ml) for 2 h at room temperature. After further washing, ExtraAvidin-Alkaline Phosphatase conjugate (Sigma Aldrich E2636, 1:400 in 1% BSA/PBS) was applied. Two hours later, wells were washed and pNPP solution (Sigma Aldrich NP2640) was added. Development was carried out in the dark at 37 °C with readings taken every 15 min at 405 nm in a Bio-Rad 680 microplate reader until the signals were fully developed.

##### Antibody Neutralization Assay

Based on initial titrations (data not shown), 1% (*v*/*v*) freshly frozen human plasma or 0.4% (*v*/*v*) mouse RA-conditional medium was used in the assays. Stimuli were mixed with neutralizing reagents or PBS as controls and pre-incubated for 1 h at room temperature to allow neutralization. The solution was added dropwise onto HPAECs in 6-well dishes, and cells were harvested after 1 h of treatment. RNA extraction, reverse transcription, and Q-PCR reaction were carried out as described above. The final concentrations of ALK1-Fc in all neutralizing experiments were 2.5 μg/ml. In the plasma neutralizing experiment, the final concentrations of anti-BMP9 antibody were 15 μg/ml (+) or 20 μg/ml (++), and anti-BMP10 antibody was applied at 15 μg/ml. In the experiment of neutralizing BMP activities from RA-conditioned medium, final concentrations of anti-BMP9 and anti-BMP10 antibodies were both at 20 μg/ml, respectively.

##### Statistical Analysis

Data are presented as mean ± S.E. Paired *t* test was used for comparison between two groups, and for multiple group comparisons, one-way ANOVA followed by Tukey's or Dunnett's post test was used when appropriate and as specified in the figure legends. *p* < 0.05 was considered statistically significant. All the analyses were performed using GraphPad Prism.

## Results

### 

#### 

##### BMP10 Prodomain Inhibits BMP10 Activity in C2C12 Cells

**FIGURE 1.**
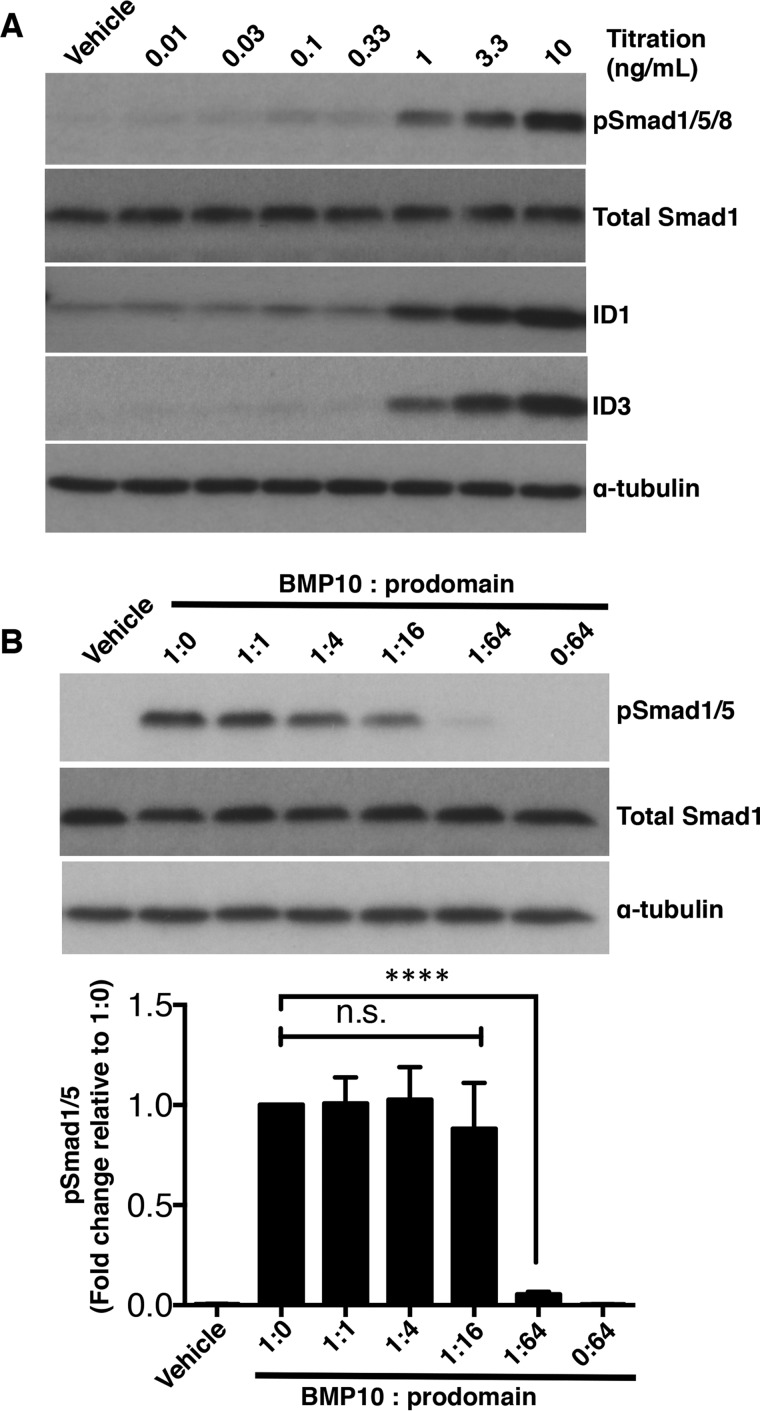
**BMP10 prodomain inhibits BMP10 activity in C2C12 cells.** *A*, titration of BMP10 GFD activity in C2C12 cells. Serum-starved C2C12 cells were treated with BMP10 GFD at increasing concentrations for 1 h, and the phosphorylation of Smad1/5 and the induction of ID1 and ID3 proteins were measured by immunoblotting analysis. Total Smad1 was used as a loading control. *B*, prodomain inhibition assay in C2C12 cells. BMP10 GFD was pre-incubated with the prodomain (molar ratio BMP10 GFD to prodomain, 1:0, 1:1, 1:4, 1:16, and 1:64) before applying to the serum-starved C2C12 cells for 1 h. Ratio 0:64 indicates the same amount of prodomain as in 1:64, but in the absence of BMP10 GFD. The remaining activity was measured by phosphorylation of Smad1/5 using immunoblotting; total Smad1 was used as a loading control. One representative blot from four repeats is shown. Below, using densitometry analysis (Image J), relative phosphorylation of Smad1/5 were corrected to total Smad1 and normalized to the sample treated with BMP10 only without the prodomain (1:0). The fold changes are expressed as mean ± S.E. *n* = 4; ****, *p* ≤ 0.0001; n.s., not significant. One-way ANOVA, Dunnett's post test.

Because the previously reported prodomain inhibition of BMP10 was carried out in C2C12 cells (8), we wished to confirm this observation prior to studies in endothelial cells. Instead of using 50 ng/ml treatment for 6 h and *ID3* gene induction as a readout (8), we chose to monitor the phosphorylation of Smad1/5 after 1 h of treatment since this event is upstream of ID gene induction and directly reflects the receptor activation by BMP10 with minimum interference from any downstream events or other signaling pathways. We also carried out immunoblotting for ID1 and ID3 proteins in the same cell lysates to establish whether the Smad1/5 phosphorylation correlates with the induction of ID proteins. We initially analyzed the dose-dependent response to the BMP10 GFD to define the concentration to use in the prodomain inhibition assay (Fig. 1*A*). We could detect Smad1/5 phosphorylation from 1 ng/ml BMP10 GFD, with robust signal detected at 10 ng/ml. No change in the total Smad1 level was observed following BMP10 treatment. Immunoblotting with anti-ID1 and anti-ID3 antibodies confirmed that this dose-dependent Smad1/5 phosphorylation is correlated with an increase in ID protein levels (Fig. 1*A*). We therefore chose to use 10 ng/ml BMP10 GFD for the prodomain inhibition assay. BMP10 GFD was pre-incubated with increasing amounts of the BMP10 prodomain as indicated in Fig. 1*B* before being applied to the serum-starved C2C12 cells. In agreement with published results, even using a different assay, we could indeed observe the inhibition by the prodomain, but only at 64-fold molar excess (Fig. 1*B*). Because it is not known whether BMP10 signaling in C2C12 cells represents any *in vivo* situation, the impact of this observation is still not fully clear.

##### BMP10 Prodomain Does Not Inhibit BMP10 Activity in Endothelial Cell Lines

**FIGURE 2.**
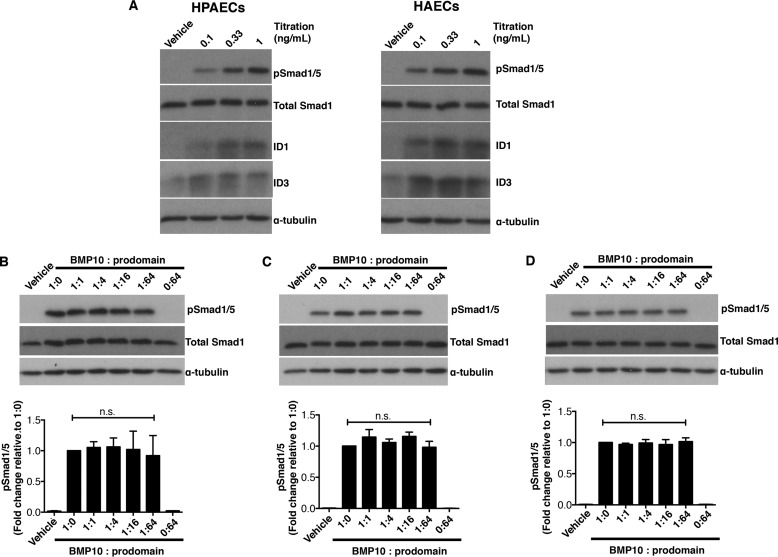
**BMP10 prodomain does not inhibit BMP10 activity in endothelial cell lines.** *A*, titration of BMP10 GFD activities in HPAECs (*left*) and HAECs (*right*). Increasing concentrations of BMP10 GFD as indicated were used to treat the serum-starved cells. After 1 h treatment, cells were harvested and the phosphorylation of Smad1/5 and the induction of ID1 and ID3 proteins were analyzed using immunoblotting. *B*, BMP10 GFD was pre-incubated with increasing amounts of BMP10 prodomain in same molar ratio as in Fig. 1*B* before applying to serum-starved endothelial cells in (*B*) HPAECs, (*C*) HAECs, and (*D*) HMEC-1. Remaining activity of BMP10 was measured by phosphorylation of Smad1/5 with total Smad1 as a loading control. One representative blot from four repeats is shown for all experiments. Prodomain inhibitions were quantified and analyzed as in Fig. 1*B* and shown below.

Because endothelial cells are the most relevant target cells to mediate BMP10 functions in adults, we tested whether the prodomain could inhibit BMP10 GFD activity in several types of endothelial cells, including HPAECs, HAECs, and HMEC-1. To establish the concentration of BMP10 GFD to use for the inhibition assay, we assessed the concentration-responses to the BMP10 GFD in HPAECs and HAECs. As shown in Fig. 2*A*, in both HPAECs and HAECs, Smad1/5 phosphorylation and ID1, ID3 protein induction were detected in response to BMP10 GFD at concentrations as low as 0.1 ng/ml with robust signals at 1 ng/ml. Again no changes were observed in the levels of total Smad1 protein following BMP10 treatment. We chose to use 1 ng/ml of BMP10 GFD and carried out the prodomain inhibition assay using the same molar excess of prodomain as described above in the C2C12 assays. In all three types of endothelial cells, we could not detect any inhibition of BMP10-mediated Smad1/5 phosphorylation by the prodomain even when applied at 64-fold molar excess (Fig. 2, *B–D*). It is difficult to envisage in any *in vivo* situations that there would be more than 64-fold excess of BMP10 prodomain present alongside its GFD, we therefore conclude that the prodomain does not inhibit BMP10 activity in endothelial cells.

##### Generation of Human pBMP10 Complex

**FIGURE 3.**
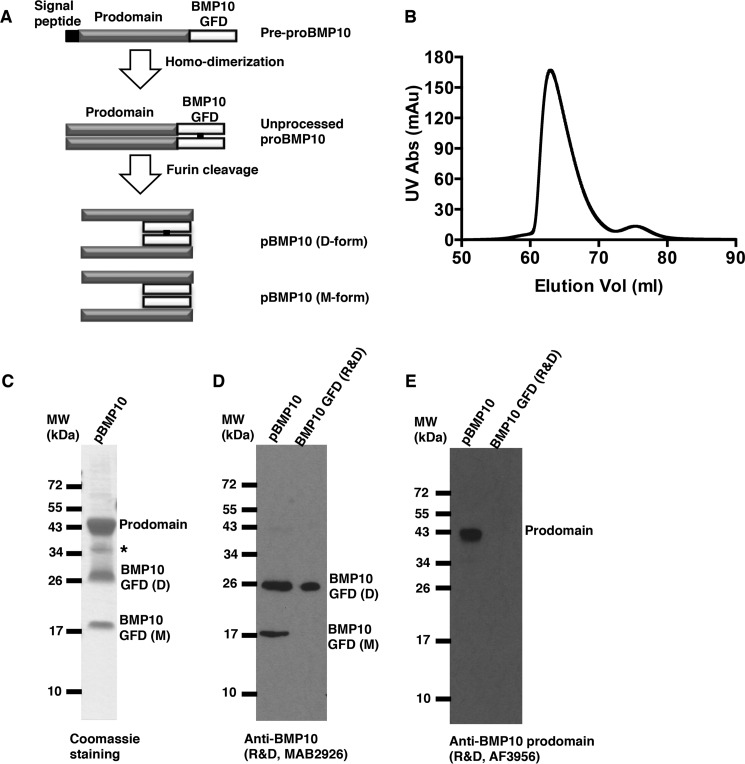
**Generation of recombinant human pBMP10.** *A*, schematic diagram of BMP10 production and processing. *B*, FPLC chromatography gel filtration trace of purified pBMP10. *C*, purified pBMP10 shown as the peak fraction from the gel filtration in *B* on a non-reducing SDS-PAGE. A *single asterisk* denotes a nonspecific protein. *D*, both D- and M-forms of BMP10 GFD could be detected by monoclonal anti-BMP10 antibody. *E,* prodomain can be detected by anti-BMP10 prodomain antibody. BMP10 GFD from R&D Systems was used as a positive control in *D* and negative control in *E*.

Having demonstrated that the prodomain does not inhibit BMP10 activities in vascular endothelial cells, we next asked whether pBMP10 complex is latent or active on these endothelial cells. To test this, we generated recombinant human pBMP10 using a mammalian expression system. Similar to all other TGFβ family members, BMP10 is synthesized as a pre-propeptide comprising a signal peptide, prodomain, and mature BMP10 GFD at the C terminus. Upon secretion, the signal peptide is removed and prodomain cleaved by furin-like proprotein convertase in the Golgi apparatus to generate the active growth factor (Fig. 3*A*). It has been shown that when recombinant BMP9 or BMP7 expressed from mammalian cells is fractionated on a non-reducing SDS-PAGE, both dimeric and monomeric BMP GFD can be detected (22, 23). We found this was also true for BMP10 (data not shown). The purified pBMP10 complex eluted under a single peak on a gel filtration column (Fig. 3*B*) and SDS-PAGE fractionation of the peak fraction showed pBMP10 complex was over 95% pure (Fig. 3*C*). Both monomeric and dimeric BMP10 GFD could be seen on a non-reducing SDS-PAGE, which are very likely to be the covalently-linked (D-form) and non-covalently linked (M-form) BMP10 dimer, as we have previously characterized for BMP9 (22), because both D- and M-forms can form a complex with the prodomain and co-elute on the size exclusion column (Fig. 3*B*). The identities of BMP10 GFD and its prodomain were confirmed by mass spectrometry in-gel trypsin digestion and peptide mapping (data not shown), as well as by immunoblotting for BMP10 GFD and its prodomain (Fig. 3, *D* and *E*). Protein N-terminal sequencing revealed amino acid residues SPIMN for prodomain and NAKGN for both BMP10 D- and M-forms, confirming the correct processing of the pBMP10 complex.

##### Prodomain-bound BMP10 Is a Highly Stable Complex

**FIGURE 4.**
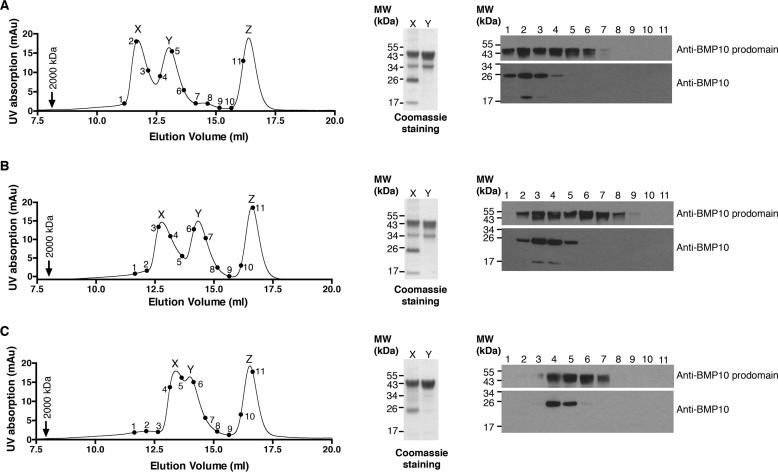
**Prodomain-bound BMP10 is a highly stable complex.** Analytical gel filtration analysis of semi-purified pBMP10 in TBS (*A*), TBS with NaCl at 1 m final concentration (*B*) or TBS with 1 m GuHCl (*C*). pBMP10 was pre-incubated in each buffer for 30 min before being loaded onto a Superdex S200 10/300 size-exclusion column pre-equilibrated in the same buffer. A control protein carbonic anhydrase (24 kDa) that does not interact with either BMP10 GFD or its prodomain was added to the pBMP10 before loading. Blue dextran (2000 kDa, *black arrow*) was run separately in each buffer to indicate the void volume. Note the protein peaks shifted slightly between the runs, potentially due to proteins interacting with the column matrix differently in different buffer systems. *Points 1–11* on the traces correspond to consecutive fractions, which were run in *lanes 1–11* of immunoblotting analyses probed with either anti-BMP10 prodomain or anti-BMP10 antibodies. *Peaks X* and *Y* were TCA precipitated, ran on a non-reducing SDS-PAGE and Coomassie Blue stained to reveal the identity of the peaks. *Peak Z* is carbonic anhydrase.

The prodomain has been shown to bind to its cognate BMP at a site that overlaps with the type II receptor binding surface (24), suggesting that the prodomain needs to be displaced to allow signaling complex formation. We tested when purified pBMP10 was subjected to different chemical conditions that are known to weaken protein-protein interactions, whether the prodomain would dissociate from BMP10 GFD. Guanidine hydrochloride (GuHCl) is a strong chaotropic agent that can interrupt hydrophobic interactions within proteins and protein complexes. It has been used extensively to denature proteins in protein unfolding studies. Some proteins start to unfold at 1 m GuHCl (25). In addition, high salt concentrations are known to affect the stabilities of protein complexes by reducing the electrostatic forces in protein-protein interactions (26, 27). To test whether the BMP10 prodomain and GFD in the pBMP10 complex would dissociate in the presence of low concentrations of chaotropic agent or high concentrations of salt, semi-purified recombinant pBMP10 (with excess prodomain) was pre-incubated in either TBS alone, or TBS with final concentrations of 1 m GuHCl or 1 m NaCl before being loaded onto an S200 10/300 gel filtration column pre-equilibrated in the same buffer. As shown in Fig. 4, pBMP10 is a highly stable complex, which did not dissociate and was eluted under a single peak in either 1 m NaCl (Fig. 4*B*, *peak X*) or 1 m GuHCl solutions (Fig. 4*C*, *peak X*). Excess prodomain was eluted under a separate peak (*peak Y* in Fig. 4, *A–C*), providing further support that prodomain was indeed in a complex with BMP10 GFD under peak X in each condition. To further demonstrate that the interaction between BMP10 and the prodomain was specific, we employed a control protein with a similar molecular weight to BMP10 GFD dimer (carbonic anhydrase, 24 kDa), which does not interact with BMP10 or its prodomain. Carbonic anhydrase was pre-mixed with pBMP10 before injection in each run. In all three buffer conditions, the control protein was eluted under a separate peak corresponding to a lower molecular weight (*peak Z* in Fig. 4, *A–C*).

##### Prodomain-bound BMP10 Is Not Latent and Is Fully Active in Multiple Endothelial Cell Lines

**FIGURE 5.**
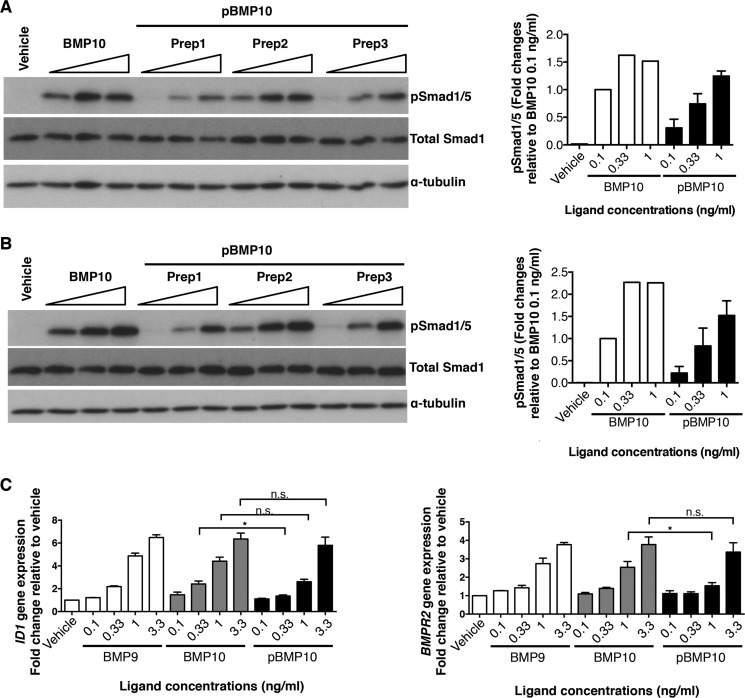
**Prodomain-bound BMP10 is active in endothelial cells.** *A* and *B,* phosphorylation of Smad1/5 in HPAECs (*A*) and HAECs (*B*) treated with 0.1 ng/ml, 0.33 ng/ml, and 1 ng/ml of BMP10 GFD or three independent preparations of pBMP10 for 1 h, detected by Smad1/5 phosphorylation in immunoblotting analysis; total Smad1 was used as a loading control. The concentrations of pBMP10 in all the cell assays refer to the concentrations of mature GFD in the pBMP10 complex. Relative phosphorylation of Smad1/5 upon treatment was measured using densitometry, corrected to total Smad1 and normalized to 0.1 ng/ml BMP10 GFD treatment condition and plotted on the *right. C,* induction of *ID1* and *BMPR2* mRNA expression by recombinant pBMP10, compared with BMP9 and BMP10 GFD. HPAECs were treated with the ligands at indicated concentrations for 8 h before samples were harvested for RNA extraction and qPCR analysis as described in “Experimental Procedures.” *n* = 3; *, *p* ≤ 0.05; *n.s.*, not significant; paired *t* test.

To test whether pBMP10 is active in endothelial cells, we quantified our in-house generated recombinant pBMP10 by immunoblotting using an anti-BMP10 antibody, with commercial BMP10 GFD as a standard (data not shown). We then treated HPAECs and HAECs with increasing concentrations of pBMP10, alongside BMP10 GFD as a control, and monitored the canonical Smad1/5 phosphorylation. As shown in Fig. 5, *A* and *B*, in both types of endothelial cells pBMP10 is slightly less active at very low concentrations, as Smad1/5 phosphorylation can be detected when cells were treated with BMP10 GFD at 0.1 ng/ml, but not with all the pBMP10 batches. However, when applied at 1 ng/ml concentration, pBMP10 induced robust Smad1/5 phosphorylation, comparable with BMP10 GFD. We further compared induction of *ID1* and *BMPR2* gene expression by BMP10 GFD and pBMP10 in HPAECs (Fig. 5*C*). These are two well-documented target genes of BMP9- and BMP10-signaling in endothelial cells. Consistent with the Smad1/5 phosphorylation results, at very low concentrations (0.33 ng/ml for *ID1* and 1 ng/ml for *BMPR2* gene inductions), pBMP10 was slightly less active than BMP10 GFD, but this difference disappeared when a slightly higher concentration of ligand was applied. These results support the conclusion that pBMP10 complex is an active ligand in endothelial cells.

##### BMPR-II ECD Can Release BMP10 from pBMP10 Complex

**FIGURE 6.**
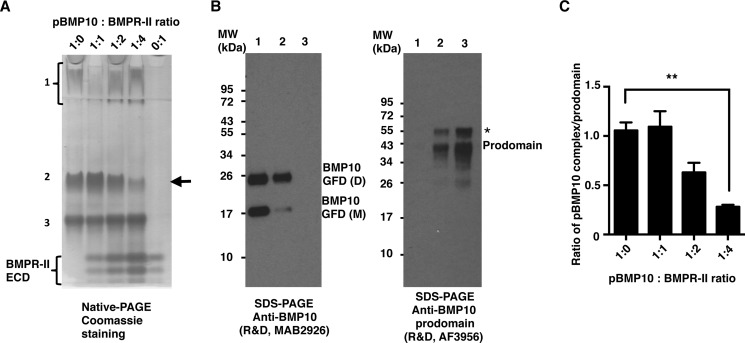
**BMPR-II ECD can release BMP10 GFD from the pBMP10 complex.** *A* and *B*, interaction between BMPR-II ECD and pBMP10 was investigated using native PAGE. Purified pBMP10 complex was run on a 10% native PAGE (*A*) either alone (1:0), or with increasing amounts of BMPR-II ECD (molar ratio of pBMP10:BMPR-II ECD, 1:1, 1:2, and 1:4). 0:1 refers to BMPR-II alone control. Prodomain-bound BMP10 complex was separated into three bands on the native PAGE. These three bands were excised from the native PAGE, run in parallel on non-reducing SDS-PAGE, and probed with either anti-BMP10 antibody or anti-BMP10 prodomain antibody (*B*). *Band 1*, which ran as a smeared band and stayed in the stacking gel, contains only BMP10 GFD, consistent with the PI of BMP10 GFD being 8.67. *Band 3* contained only the prodomain (PI = 4.54) and ran fastest on the SDS-PAGE. *Band 2* contained both BMP10 GFD and the prodomain, hence is the pBMP10 complex. The *arrow* in *A* points to the decrease in the pBMP10 complex upon adding increasing amounts of BMPR-II ECD. A *single asterisk* in *B* denotes either a nonspecific protein in the protein prep or a differentially processed BMP10 prodomain. *C*, changes of pBMP10 complex in *A* were quantified as the ratio of the pBMP10 complex/prodomain alone to normalize for the loading. *n* = 4, one-way ANOVA, Dunnett's post test. **, *p* ≤ 0.01.

Because the pBMP10 complex is active on endothelial cells, one possibility is that the contact of pBMP10 with the receptors can release BMP10 GFD from the complex. It has been shown for BMP7 and BMP9 that the prodomain competes for the binding of the type II receptors, not the type I receptors (24, 28). We therefore tested whether BMPR-II ECD can displace the prodomain from the pBMP10 complex. Using native PAGE analysis, we found that the pBMP10 complex migrated as three bands (Fig. 6*A*, 1:0). Immunoblots of the proteins eluted from the three bands (Fig. 6*B*) revealed their identities as BMP10 GFD alone (band 1), pBMP10 complex (band 2), and prodomain alone (band 3). Interestingly, when increasing amounts of BMPR-II ECD were added, pBMP10 complex decreased in a dose-dependent manner (Fig. 6*A*, *black arrow*). To confirm that this was not due to the variation in the amount of protein loaded onto the native PAGE, we repeated the experiment four times and plotted the ratio of pBMP10 : prodomain (Fig. 6*C*). We observed a reduction of pBMP10 complex when BMPR-II ECD was present at 2-fold excess, and this reduction reached statistical significance at 4:1 ratio of BMPR-II ECD : prodomain, confirming that BMP type II receptor can indeed compete for prodomain binding and release the BMP10 GFD from the pBMP10 complex *in vitro*.

##### BMP10 Derived From Atrium or Plasma Is Fully Active

**FIGURE 7.**
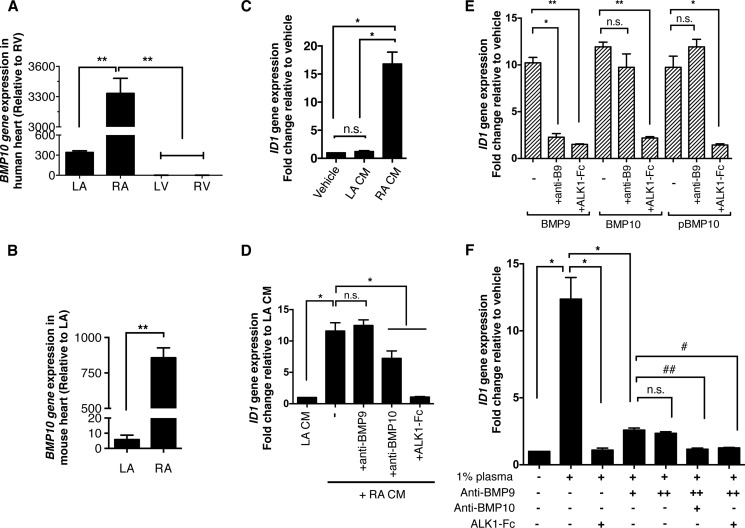
**BMP10 derived from atrium or plasma is fully active.** *A*, BMP10 mRNA expression in human heart tissues. *n* = 3. **, *p* ≤ 0.01; one-way ANOVA, Tukey's post test. *B*, BMP10 mRNA expression in mouse heart tissues. *BMP10* expression is significantly higher in RA than LA in mouse. *n* = 4, paired *t* test; **, *p* ≤ 0.01; *C*, BMP activity could be detected in the conditioned medium of cultured mouse RA. Conditioned medium from LA or RA was applied to serum-starved HPAECs (both at 5% *v*/*v*), and the BMP activity was measured by the induction of *ID1* gene expression. No BMP activity can be detected from LA-conditioned medium (*LA CM*), while significant level of *ID1* gene induction activity can be detected in the RA-conditioned medium (*RA CM*). *n* = 3, * *p* ≤ 0.05; n.s., not significant. One-way ANOVA, Tukey's post test; *D*, identification of BMP activity in RA CM. The *ID1*-induction activity from RA CM (0.4% *v*/*v*) could not be inhibited by anti-BMP9 antibody (at 20 μg/ml), but can be partially inhibited by anti-BMP10 antibody (at 20 μg/ml), and very effectively inhibited by ALK1-Fc (at 2.5 μg/ml). *n* = 3, *, *p* ≤ 0.05; *n.s*., not significant. One-way ANOVA, Dunnett's post test; *E*, control experiments showed that anti-BMP9 antibody (at 10 μg/ml) could specifically neutralize BMP9 activity very effectively, but not the activity of BMP10 or pBMP10, whereas ALK1-Fc (at 2.5 μg/ml) can inhibit both BMP9 and BMP10 activity very effectively. The concentrations of BMP9, BMP10, and pBMP10 used in this assay were all 1 ng/ml. *n* = 3, one-way ANOVA for each BMP ligand group, Dunnett's post test, **, *p* ≤ 0.01; *, *p* ≤ 0.05; *n.s*., not significant; *F*, BMP10 activity can be detected in human plasma. Freshly frozen human plasma was used to treat serum-starved HPAECs (1% *v*/*v* final concentration), and BMP activity was measured by *ID1* gene induction. All the *ID1*-induction activity from 1% plasma can be completely inhibited by ALK1-Fc (at 2.5 μg/ml) alone, suggesting that all the *ID1*-gene induction activity in 1% plasma was due to BMP9 and BMP10. While most of this activity can be inhibited by anti-BMP9 antibody (at 15 μg/ml), the residual *ID1*-induction activity cannot be inhibited by additional amounts of anti-BMP9 antibody (at 20 μg/ml). It can be only inhibited by either anti-BMP10 antibody (at 15 μg/ml) or ALK1-Fc (at 2.5 μg/ml), suggesting the residual *ID1*-induction activity is due to BMP10. *n* = 3, one-way ANOVA, Dunnett's post test. *, *p* ≤ 0.05; #, *p* ≤ 0.05; ##, *p* ≤ 0.01; *n.s*., not significant.

Having shown that recombinant pBMP10 complex is fully active, we hypothesized that RA-secreted BMP10 is also active and not in the latent form. We first of all confirmed the specific expression of BMP10 in adult heart tissue. In human adult heart, no BMP10 expression could be detected in left or right ventricles (Fig. 7*A*). BMP10 expression could be detected in the LA, but the expression level was nearly 10 times higher in the RA. We then confirmed the BMP10 expression in left and right atria from adult mice, and found that the expression level of BMP10 in the RA was about 170-fold higher than that in the LA (Fig. 7*B*).

To ascertain whether the RA-secreted BMP10 is active or not, we tested BMP10 activity secreted from *ex vivo* cultured mouse RA, with LA cultured alongside as a negative control. As shown in Fig. 7*C*, strong *ID1* gene induction activities could be detected in HPAECs treated with conditioned medium from the RA, but not in that from the LA. This *ID1*-induction activity was completely inhibited by ALK1-Fc, partially inhibited by anti-BMP10 antibody, but not at all by anti-BMP9 antibody (Fig. 7*D*). Control experiments showed that anti-BMP9 antibody could effectively neutralize BMP9 activity but it could not inhibit either BMP10 or pBMP10 activity, whereas ALK1-Fc could block both the BMP9 and BMP10 signaling activities (Fig. 7*E*). Although we could not detect any neutralizing activity for BMP10 GFD or pBMP10 in this assay using the commercially available anti-BMP10 antibodies (data not shown), we could observe partial inhibition using anti-BMP10 antibody when we used a smaller volume of the RA-conditioned medium in the signaling assay (Fig. 7*D*). These data strongly suggested that the *ID1* gene-induction activity in the RA-conditioned medium was indeed due to BMP10, not BMP9. This is in agreement with previous reports that BMP9 is mostly expressed in the liver and no BMP9 expression was detected in the heart (14, 29). Next we investigated whether we could detect any BMP10 activity in human plasma (Fig. 7*F*). Freshly frozen plasma (at 1% final concentration) potently induced *ID1* gene expression in HPAECs, and this activity was completely inhibited by ALK1-Fc, suggesting that all the ID1-induction activity in 1% plasma was attributed to BMP9 and BMP10. While most of this ID1-induction activity was inhibited by anti-BMP9 antibody, the remaining activity could not be inhibited by a higher concentration of anti-BMP9 antibody, but was inhibited effectively by either anti-BMP10 antibody or ALK1-Fc, indicating that this remaining ID1 gene induction activity was due to BMP10.

##### RA-derived Active BMP10 Exists as the pBMP10 Complex

**FIGURE 8.**
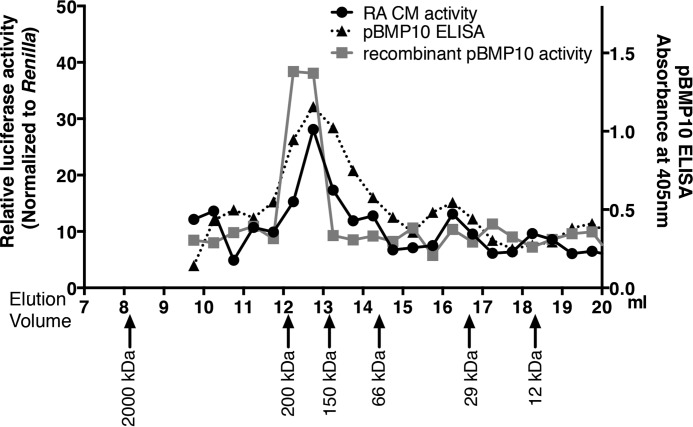
**Right-atrium-secreted active BMP10 is in the prodomain-bound form.** Proteins from RA-conditioned medium were separated by gel filtration chromatography, and the BMP activities in the fractions were measured using the BRE-luciferase assay as described in “Experimental Procedures.” The relative BRE-luciferase activities in the fractions were plotted against their elution volumes (*black line with circle symbols*). The same process was repeated with diluted, purified recombinant human pBMP10, and the relative BMP activities in the fractions were also measured and plotted (*gray line with square symbols*). In addition, a pBMP10-specific ELISA was carried out to measure the proteins in the fractions from RA-conditioned medium gel filtration. Data were presented as optical densities (absorbance at 405 nm, right axis, *dotted line with triangular symbols*). Proteins from Gel Filtration Calibration Kit were run under identical conditions, and *arrows* below showed the elution volumes of the protein standards.

Next we questioned whether the BMP10 activity from natural source was in the form of BMP10 GFD or pBMP10 complex. Plasma contains both BMP9 and BMP10, which are difficult to separate, whereas conditioned medium from the RA contains only BMP10. Therefore, we chose to fractionate the RA-conditioned medium using S200 size-exclusion chromatography to separate proteins of different sizes. We then monitored the BMP activities from the fractions using BRE-luciferase activity assays in ALK1-transfected C2C12 cells, since the fractions could only contain BMP10 activity (Fig. 7*D*). As shown in Fig. 8, a control experiment showed that when recombinant pBMP10 was applied to the gel filtration column, BRE-luciferase activity could be detected in fractions with a peak at around 12.5 ml, corresponding to a protein or protein complex with a molecular weight of 138–155 kDa according to the calibration standards. The expected molecular weight of a dimeric pBMP10 complex, calculated from amino acid compositions, is 92 kDa. The higher than expected molecular weight could be due to glycosylation as there are two predicted glycosylation sites in each prodomain, hence four glycosylation sites in the dimeric pBMP10 complex. In addition, it has recently been shown by electron microscopy that pBMP9 complex (and pBMP7 complex) adopts an elongated open conformation (28). If pBMP10 complex adopts a similar open conformation, it will also contribute to the apparent higher molecular weight on a size exclusion column. When RA-conditioned medium was applied to the S200 column under identical conditions, peak BRE-luciferase activity was detected at around 12.7 ml, indicating that BMP10 activity in RA-conditioned medium was due to a protein or protein complex with a similar molecular weight to the pBMP10 complex.

To confirm that BMP10 activity in the RA-conditioned medium was indeed in the form of pBMP10 complex, we subjected the fractions from RA-conditioned medium gel filtration to a pBMP10-specific ELISA assay, using anti-BMP10 monoclonal antibody as the capture antibody, and biotinylated anti-prodomain antibody as the detecting antibody. This ELISA will only show positive signals in fractions containing pBMP10 complex. As shown in Fig. 8, the profile of ELISA signals mirrored that of the BRE-luciferase activities, with the peaks aligned perfectly, supporting the hypothesis that active BMP10 secreted from mouse RA was indeed in the prodomain-bound complex.

## Discussion

In this study, we demonstrated that exogenous addition of the BMP10 prodomain did not inhibit BMP10 GFD activity in three endothelial cell lines, including primary endothelial cells. Using the recombinant human pBMP10 complex overexpressed in mammalian cells and purified under native conditions, we showed that pBMP10, contrary to being latent, was fully active in inducing Smad1/5 phosphorylation and the *ID1* and *BMPR2* gene expression in multiple endothelial cell lines. In addition, we have demonstrated BMP10 activity in freshly frozen human plasma. Finally, we show evidence that the right atrium secretes active BMP10 as the prodomain-bound complex.

Differential expression of BMP receptors, especially the high affinity receptors, on the cell surface can confer different sensitivity to diverse BMPs. For example, the high affinity type I receptors for BMP4 and BMP9 are ALK3 and ALK1 respectively. Relative to smooth muscle cells (SMCs), endothelial cells express higher levels of ALK1 and much lower levels of ALK3 (30, 31). This would predict that BMP9 signals preferentially in endothelial cells and BMP4 in SMCs. Indeed, a recent publication reports a side-by-side comparison between BMP4 and BMP9 signaling in HPAECs and pulmonary artery SMCs (21). BMP9 induced strong Smad1/5 phosphorylation in endothelial cells at concentrations as low as 0.1 ng/ml, but only induced weak Smad1/5 phosphorylation at 1 and 10 ng/ml in SMCs. Similarly, Smad1/5 phosphorylation induced by BMP4 treatment can be detected at 0.1 ng/ml in SMCs, but not at 100 ng/ml in endothelial cells (21). Using physiologically relevant cells to address the function of a specific BMP allows the BMP ligand to be tested at close to physiological concentrations, and in the correct receptor context. Endothelial cells are the most physiologically relevant cell types for investigating BMP9/10 function since BMP9/10 have been proposed to act as vascular quiescence factors that signal via ALK1 in endothelial cells (20, 32). In addition, the localized expression and secretion of BMP10 from the right atrium means that the pulmonary vascular endothelium is likely a major downstream target for this ligand. Hence, we focused on using endothelial cells to investigate BMP10 activity. Previous reports showing that the prodomain inhibits BMP10 activity were conducted in C2C12 cells without ALK1 transfection. Although we have recapitulated this result in the present study, we clearly demonstrated that this was not the case in endothelial cells. Under physiological conditions, the roles and signaling pathways of BMP10 in embryonic development may be different from that in adult life. It was elegantly shown that BMP10 has two distinct functions during development; one is to support early vascular development, which can be substituted by BMP9, the other is to regulate heart development that cannot be substituted by BMP9 (16). It is tempting to speculate that BMP10 may act on a different cell type to exert its role in heart development, and that this is likely to be mediated through a different set of receptor complexes. Thus the inhibitory role of the prodomain on BMP10 activity may still hold true in other cellular contexts.

Until now, the reports on whether BMP10 circulates and is active have been inconclusive. On the one hand, circulating BMP10 from mouse and human sera can be detected by ELISA (12) as well as by pull-down coupled with proteomics (13), but its activity has not been detected in two previous reports (15, 17). Again, this absence of detectable BMP10 activity in those reports could be due to the cell types used for the activity assays. In one report, Herrera *et al.* found that in C2C12 reporter cells, the lower sensitivity for BMP9 was 0.1 ng/ml and for BMP10 was 1 ng/ml (15). Clearly this C2C12 assay would not be able to detect BMP10 activity if it is present in the plasma at concentrations lower than 1 ng/ml, and we have shown here that pBMP10 complex is active at 0.33 ng/ml in inducing Smad1/5 phosphorylation in endothelial cells. In another study, NIH-3T3 cells were transfected with ALK1 to enhance their sensitivity to BMP9 and BMP10. Although anti-BMP9 antibody abolished the activity of recombinant BMP9, it did not completely inhibit the serum-induced BRE-stimulating activity (17). Unfortunately, neither anti-BMP10 antibody nor ALK1-Fc was tested to further inhibit the residual BRE-luciferase activity. Consistent with this latter study, we observed that the BMP activity in human plasma measured by *ID1* mRNA induction in HPAECs was largely contributed by circulating BMP9, which could be effectively inhibited by an anti-BMP9 antibody. In addition, we demonstrated that the residual activity was only inhibited by anti-BMP10 antibody or by ALK1-Fc, not by further addition of anti-BMP9 antibody. By monitoring *Smad6* induction in human umbilical vein endothelial cells, Chen *et al.* detected BMP10 activity in mouse serum (16). To our knowledge this is the first time that the presence of circulating BMP10 activity in human plasma has been demonstrated.

Our finding that RA-secreted BMP10 is active on endothelial cells implies that BMP10 may play an active hitherto unrecognized role in adult life. BMP9 and BMP10 are the only two known ligands activating the ALK1-mediated pathways and control the expression of a similar set of genes (12, 20). They signal redundantly *in vivo* and BMP10 can substitute BMP9 for postnatal retinal vascular remodeling in *BMP9*^−/−^ mice (12, 16). BMP9 is a vascular quiescence factor in the vascular endothelium (17) and BMP10 has been shown to mediate flow-dependent arterial quiescence in zebrafish (32). Since RA-secreted BMP10 will first enter the right ventricle and then pulmonary circulation, BMP10 may contribute to the vascular quiescence in the pulmonary endothelium. It is interesting to note that mutations in the *BMPR2* gene, which encodes the major type II receptor for the large family of BMPs, is the most common genetic cause of PAH, a disease characterized by the extensive remodeling of pulmonary arteries, right ventricular hypertrophy and heart failure (33). Most importantly, selective enhancement of the endothelial BMPR-II pathway with BMP9 can reverse established PAH in several animal models (21). It would be interesting to test whether BMP10-mediated signaling plays a protective role in the pulmonary circulation, and since pBMP10 complex is an active and redundant ligand to BMP9, pBMP10 could also have therapeutic potential for treating PAH.

Questions remain regarding the role of the prodomain in regulating BMP signaling. For example, BMP9 and BMP10 share 64% sequence identity in the GFD, but only 29% in their prodomains. It is possible that this sequence variation confers temporal and spatial signaling specificity to BMP9 and BMP10. In addition, quantitative ELISA binding measurements for BMP7 and BMP9 show that the prodomain has little effect on the affinities for type I receptor, but can alter both the EC_50_ and the selectivity of type II receptor binding to BMPs (24, 28). Such selectivity of different type II receptors, conferred by the prodomain, is yet to be demonstrated in cell signaling and functional assays. It is interesting to note that, similar to our observation that the prodomain can inhibit BMP10 activities in C2C12 cells but not in endothelial cells and that prodomain-bound BMP10 is fully active, it has recently been shown that the prodomain of BMP9, albeit at a large molar excess, can also inhibit BMP9 activity in C2C12 cells (28) despite that prodomain-bound BMP9 is fully active in multiple cell types (14, 34). For the question of whether and how the BMP prodomain could confer latency to the GFD under certain conditions, it was recently proposed that the pBMP9 complex could adopt both cross-armed, latent, and open-armed, non-latent conformations (28). Cross-armed conformation would resemble the latent TGFβ structure. Integrin is a key activator of latent TGFβ, acting through binding to the RGD sequence in the TGFβ prodomain. However, there is no RGD sequence in the prodomain of either BMP9 or BMP10. Whether pBMP10 or pBMP9 can adopt a cross-armed latent conformation is still to be tested. A crystal structure of a pBMP complex in a crossed-armed conformation will provide the ultimate answer to this hypothesis.

## Author Contributions

H. J., R. M. S., and W. L. designed the experiments. H. J., R. M. S., A. L., and Z. W. performed the experiments and collected data. P. D. U. and A. P. D. collected the human tissues and performed the experiments on human tissue. H. J., R. M. S., and W. L. analyzed and interpreted the data. W. L. conceived the idea. W. L. and N. W. M. directed the study and wrote the report.

## Footnotes

7 The abbreviations used are:

BMP10: bone morphogenetic protein 10
pBMP10: prodomain-bound BMP10 complex
TGFβ: transforming growth factor β
ALK1: activin receptor-like kinase 1
BMPR-II: BMP receptor type II
ActR-IIA(B): activin receptor type-2A(B)
GFD: growth factor domain
ID1(3): inhibitor of DNA-binding 1 (3) gene
PAH: pulmonary arterial hypertension
HPAEC: human pulmonary artery endothelial cells
HAECs: human aortic endothelial cells
proBMP10: full-length unprocessed BMP10 propeptide
ECD: extracellular domain
RA: right atrium
LA: left atrium
RV: right ventricle
LV: left ventricle
q-PCR: quantitative PCR
B2M: β-2-microglobulin
BRE: BMP-responsive element
GuHCl: guanidine hydrochloride
SMC: smooth muscle cells.
